# Digital Smile Design and Patient-Centered Outcomes in Esthetic Restorative Dentistry: A Systematic Review

**DOI:** 10.7759/cureus.98084

**Published:** 2025-11-29

**Authors:** Loulwa K Alwabel, Wateen M Alabduljabar, Lojjin K Alawfi, Nura K Al Johani, Laura E Alharbi, Mohammed A Kharif, Fatimah D Albahrani, Mutaz B Alarjani, Mazen S Alzahrani, Abeer A Alshammari, Ashwaq Alashjai, Laila Alanaz

**Affiliations:** 1 Prosthodontics, College of Medicine and Dentistry, Riyadh Elm University, Riyadh, SAU; 2 General Dentistry, Princess Nourah bint Abdulrahman University, Riyadh, SAU; 3 General Dentistry, Taibah University, Medina, SAU; 4 Dentistry, King Saud University, Riyadh, SAU; 5 General Dentistry, College of Medicine and Dentistry, Riyadh Elm University, Riyadh, SAU; 6 Dentistry, Al Baha University, Al Baha, SAU; 7 General Dentistry, Vision College, Riyadh, SAU; 8 Dentistry, King Abdullah bin Abdulaziz University Hospital (KAAUH), Riyadh, SAU; 9 Dentistry, Private Practice, Riyadh, SAU

**Keywords:** digital smile design, esthetic restorative dentistry, patient-centered outcomes, patient satisfaction, quality of life

## Abstract

Digital Smile Design (DSD) has become an increasingly valuable tool in esthetic restorative dentistry, offering digital visualization, improved communication, and enhanced precision during treatment planning. This systematic review evaluated the influence of DSD on patient-centered outcomes, including satisfaction, esthetic perception, quality of life, and psychosocial impact. A comprehensive search of PubMed/MEDLINE, ScienceDirect, Google Scholar, and the Cochrane Library was performed from inception to August 2025, following Preferred Reporting Items for Systematic Reviews and Meta-Analyses (PRISMA) guidelines. Studies were eligible if they assessed DSD within esthetic restorative procedures and reported patient-centered outcomes. Seven studies met the inclusion criteria, encompassing randomized controlled trials, observational studies, and qualitative analyses from diverse clinical settings. Sample sizes ranged from small qualitative cohorts to large randomized trials. Across all included studies, DSD consistently improved patient satisfaction, treatment acceptance, communication, and perceived predictability compared with conventional approaches. Quantitative evidence demonstrated significantly higher satisfaction scores and superior esthetic and functional ratings in DSD-guided treatments. Studies with follow-up data also reported stable long-term outcomes and improved self-confidence among patients. Despite these promising results, the evidence base remains limited by heterogeneity in study designs, varied outcome measures, and small sample sizes in some studies. Overall, DSD appears to enhance patient-centered outcomes in esthetic restorative dentistry, but further high-quality, longitudinal research is needed to validate its long-term clinical and psychosocial benefits.

## Introduction and background

Aesthetics in restorative dentistry go beyond mere function; achieving a smile that meets patient expectations has significant impacts on satisfaction, self-esteem, and broader quality of life. Traditional analog methods (manual impressions, wax-ups, etc.) often suffer from limitations in predictability, patient involvement, and communication of expected outcomes [1,2]. Digital Smile Design (DSD) emerged to address these shortcomings and represents a pivotal evolution within the broader history of digital planning in dentistry, following early advances in three-dimensional (3D) imaging, digital model analysis, and computer-assisted orthodontic planning [3]. DSD integrates digital photography, standardized photo protocols, intraoral and extraoral scanning, computer-aided design (CAD)-based analysis, and virtual mock-ups to enhance precision during esthetic planning. A typical DSD workflow includes high-resolution facial photographs aligned with reference planes, intraoral scans or digital impressions, CAD simulation of the proposed smile, and fabrication of 3D-printed or milled mock-ups that allow patient visualization before irreversible procedures.

Evidence shows that DSD facilitates clearer communication, enhances patient participation in treatment planning, and improves alignment between expected and achieved outcomes [4]. When digital impressions are used in DSD workflows, accuracy has been shown to be comparable or superior to conventional impressions, further improving predictability [5]. Multiple studies show that DSD facilitates better communication between clinicians and patients, enhances patient participation in treatment planning, improves patient satisfaction, and helps align treatment results with patient expectations [6]. For example, a randomized controlled trial comparing DSD vs conventional smile design found significantly higher satisfaction scores and improved restoration esthetics, fit, and occlusion in the DSD group [7]. Narrative reviews also point to higher acceptance rates, reduced chair time, and better esthetic outcomes when DSD is used [4]. Furthermore, patient-centered outcomes such as oral health-related quality of life (OHRQoL) may also improve with digital planning. For example, a recent study reported enhanced OHRQoL and satisfaction in patients treated with digitally planned aligner therapy compared to traditional appliances [8]. Similarly, restorative procedures guided by digital workflows-such as ultrathin ceramic veneers-have shown high esthetic success and positive patient experiences [9]. Moreover, observational studies and surveys indicate that laypersons and dental professionals tend to prefer DSD-derived designs when evaluating esthetic features such as smile harmony, tooth proportion, and symmetry [10]. A comparative study in aesthetic restoration of anterior teeth showed digital crown extension guides producing superior clinical outcomes vs traditional methods, with improved esthetic and functional restoration [11]. Studies from Indonesia also report high levels of long-term patient satisfaction, enhanced self-confidence, and stable esthetic and functional outcomes after DSD-guided treatments [12]. Despite these promising findings, there remain gaps: many studies use diverse outcome measures, there is limited long-term follow-up in some cases, and psychosocial outcomes have not been uniformly assessed. Given this, the present systematic review aims to synthesize current evidence on DSD in esthetic restorative dentistry, focusing specifically on patient-centered outcomes such as satisfaction, esthetic perception, quality of life, and psychosocial impact.

## Review

Methodology

Protocol and Registration

This systematic review was conducted in accordance with the Preferred Reporting Items for Systematic Reviews and Meta-Analyses (PRISMA) guidelines [13]. The aim of this review was to evaluate the impact of Digital Smile Design on patient-centered outcomes in esthetic restorative dentistry.

Eligibility Criteria

Studies were considered eligible if they evaluated the use of DSD in esthetic restorative dentistry and assessed patient-centered outcomes, including satisfaction, quality of life, esthetic perception, or psychosocial impact. Both randomized controlled trials (RCTs) and observational studies were included. Case reports, expert opinions, and narrative reviews were excluded. Only studies published in English were considered to ensure consistency of interpretation.

Information Sources and Search Strategy

A comprehensive electronic search was performed across PubMed/MEDLINE, ScienceDirect, Google Scholar and the Cochrane Library. All databases were searched from inception to August 30, 2025. The following keywords and Medical Subject Headings (MeSH) were used in combination with Boolean operators: “Digital Smile Design”, “esthetic restorative dentistry”, “aesthetic dentistry”, “patient satisfaction”, “quality of life”, and “patient-centered outcomes”. The search strategy was further refined through manual screening of the reference lists of eligible studies and relevant review articles to identify additional records. A full, database-specific, reproducible search strategy is provided in Appendix Table 1, as recommended by PRISMA guidelines.

Study Selection

**Figure 1:**
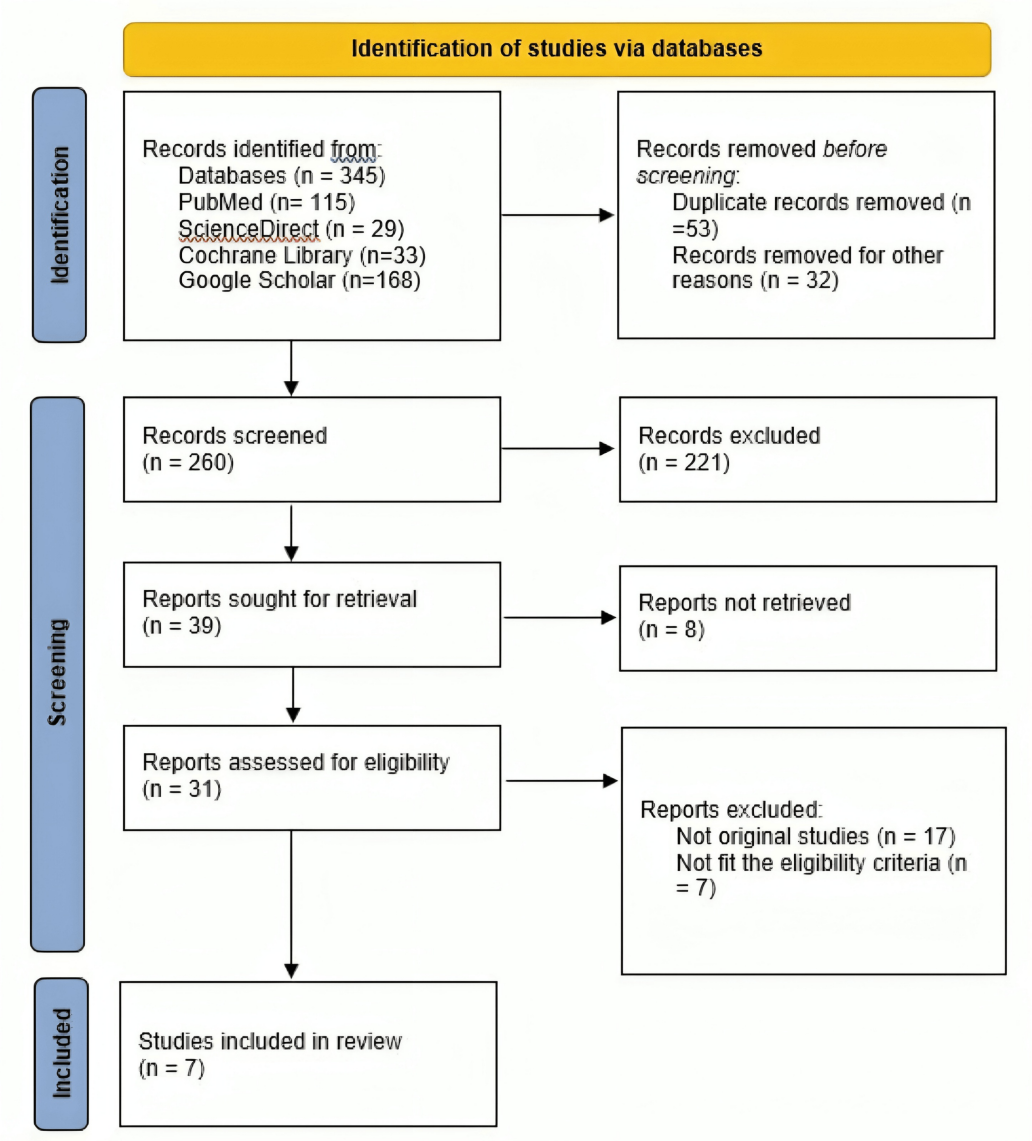
PRISMA flowchart showing the study selection process PRISMA: Preferred Reporting Items for Systematic Reviews and Meta-Analyses

All identified articles were imported into a reference management software, and duplicates were removed. Two independent reviewers screened titles and abstracts for eligibility. Full texts of potentially relevant studies were then retrieved and assessed according to the predefined inclusion and exclusion criteria. Disagreements between reviewers were resolved through discussion or consultation with a third reviewer. The selection process was documented in a PRISMA flow diagram (Figure 1).

Data Extraction

Data were extracted independently by two reviewers using a standardized data extraction form. Extracted information included: authorship, year of publication, country of study, study design, sample size, characteristics of participants, type of restorative procedure, method of Digital Smile Design application, comparator interventions (if applicable), outcome measures assessed, and key findings related to patient-centered outcomes.

Meta-analysis

A quantitative meta-analysis was not conducted due to substantial clinical and methodological heterogeneity among the included studies, which precluded meaningful statistical pooling. The studies varied considerably in design (randomized controlled trials, observational studies, qualitative research), sample characteristics, types of esthetic restorative procedures, and Digital Smile Design protocols. Crucially, the outcome measures for patient-centered results were incompatible, utilizing different scales (e.g., Visual Analog Scale, Likert scales), assessment timepoints, and definitions of success. These differences prevented meaningful statistical pooling and comparison of effect sizes. Therefore, a qualitative synthesis was deemed the most appropriate approach for this review.

Data Synthesis

Given the methodological diversity of the included studies, a narrative synthesis approach was employed. The studies demonstrated heterogeneity in design, populations, Digital Smile Design protocols, types of restorative procedures, and the patient-centered outcome measures used. Because these variations prevented meaningful statistical pooling, the findings were synthesized descriptively. Studies were grouped according to the type of esthetic intervention and the specific patient-centered outcomes assessed. The narrative synthesis focused on identifying common patterns, comparing results across study designs, and highlighting consistent themes related to the impact of Digital Smile Design on patient satisfaction, esthetic perception, and overall treatment experience.

Quality Assessment

The methodological quality and risk of bias of the included studies were assessed independently by two reviewers using appropriate validated tools based on study design. Randomized controlled trials were evaluated using the Cochrane Risk of Bias 2.0 (RoB-2) tool, which assesses bias arising from the randomization process, deviations from intended interventions, missing outcome data, measurement of outcomes, and selective reporting [14]. Observational studies (cross-sectional, retrospective, and mixed-methods designs) were appraised using the Newcastle-Ottawa Scale (NOS) adapted for non-randomized studies, evaluating selection of participants, comparability of study groups, and outcome assessment [15]. Qualitative studies were assessed using the Critical Appraisal Skills Programme (CASP) Qualitative Checklist, which examines clarity of aims, appropriateness of methodology, recruitment strategy, data collection, researcher reflexivity, ethical considerations, and rigor of analysis. Each study was independently scored by two reviewers, and disagreements were resolved through discussion. Overall, the quality of the included studies ranged from moderate to high. The randomized controlled trial demonstrated low risk of bias in most domains; however, several observational studies showed limitations related to participant selection, lack of control groups, and variability in outcome measurement tools. The qualitative study demonstrated good methodological rigor but lacked detailed reporting on reflexivity.

Results

A total of 345 records were initially identified through database searching, of which 53 duplicates and 32 irrelevant records were removed before screening. After title and abstract screening of 260 records, 221 were excluded, leaving 39 articles for full-text assessment. Of these, 31 studies were assessed for eligibility, and ultimately seven studies met the inclusion criteria and were incorporated into the qualitative synthesis. A detailed appraisal of the randomized controlled trial [7] using the RoB-2 tool is presented in Table 1.

**Table 1:** Quality assessment of randomized controlled trials using ROB-2 tool

Study	Randomization Process	Deviations From Intended Interventions	Missing Outcome Data	Measurement of Outcomes	Selection of Reported Results	Overall Risk of Bias
Luniyal et al. (2023) [7]	Low risk	Low risk	Low risk	Low risk	Low risk	Low Risk

The quality assessment of observational studies [12,16-18] using the Newcastle-Ottawa Scale (NOS) is summarized in Table 2.

**Table 2:** Quality assessment of observational studies using Newcastle–Ottawa scale (NOS)

Study	Selection (0–4)	Comparability (0–2)	Outcome (0–3)	Total Score (0–9)	Quality Rating
Babaei et al. (2025) [16]	3	1	2	6/9	Moderate
Sari et al. (2024) [12]	4	1	3	8/9	High
Cattoni et al. (2016) [17]	3	1	2	6/9	Moderate
Xuebo and Sutanto (2025) [18]	3	1	2	6/9	Moderate

The methodological appraisal of the qualitative study [19] based on the CASP Qualitative Checklist is shown in Table 3.

**Table 3:** Quality assessment of qualitative study using CASP checklist CASP:  Critical Appraisal Skills Programme.

CASP Domain	Tallarico et al. (2025) [15]
Clear aims	Yes
Appropriate design	Yes
Appropriate recruitment	Yes
Data collection adequate	Yes
Reflexivity discussed	Partial
Ethical issues addressed	Yes
Rigorous analysis	Yes
Clear statement of findings	Yes
Overall Quality	High

The included studies comprised a mix of randomized controlled trials [7], observational studies [12,16-18], qualitative analyses [19], and a case report [20], conducted across diverse geographical contexts including Iran, India, Brazil, Indonesia, Italy, and Spain. Sample sizes ranged from small-scale qualitative studies involving five participants to large randomized controlled trials with up to 150 patients. The restorative interventions evaluated included veneers, crowns, composite restorations, gingivoplasty, and full smile design procedures, all integrated with Digital Smile Design methodologies. This is summarized in Table 4.

**Table 4:** Summary characteristics of included studies DSD: Digital smile design, VAS: Visual analogue scale, CAD: Computer-aided design, CAM: Computer-aided manufacturing.

Author (Year)	Country	Study Design	Sample Size & Characteristics	Restorative Procedure	Digital Smile Design Application	Comparator (if any)	Patient-Centered Outcomes Measured	Key Findings
Babaei et al. (2025) [16]	Iran	Descriptive-analytical, cross-sectional	20 participants; 50% male, 50% female; seeking smile design	Smile design	Visagism, Proportional, and Stepwise Comprehensive DSD methods	None	Satisfaction via questionnaire (7 items, 5-point Likert scale)	High satisfaction with all DSD methods; no significant differences between methods. Visagism and Proportional slightly outperformed Stepwise Comprehensive.
Luniyal et al. (2023) [7]	India	Randomized controlled trial	150 patients; DSD group (n=75), conventional group (n=75)	Smile enhancement	Intraoral scans, CAD, 3D simulations	Conventional methods (manual impressions, wax-ups)	Satisfaction (VAS 0-100), treatment outcomes (fit, occlusion, esthetics)	DSD group had higher satisfaction (87.2 vs. 81.5) and better treatment outcomes (92% vs. 78% excellent).
Meereis et al. (2016) [20]	Brazil	Case report with follow-up	1 female, age 19, with enamel hypoplasia	Gingivoplasty, bleaching, porcelain laminate veneers	DSD for planning and communication	None	Satisfaction, communication, long-term esthetic/functional outcomes	High patient satisfaction; excellent outcomes at 2 years; improved communication and predictability.
Sari et al. (2024) [12]	Indonesia	Retrospective study	75 patients; mean age 34.5; 56% female	Veneers, crowns, composite restorations	DSD with virtual design and mock-ups	None	Satisfaction (5-point Likert scale), long-term functionality, stability, esthetics	High satisfaction (mean scores 4.6-4.9/5); 92% satisfied with smile; 88% improved confidence; excellent long-term outcomes.
Cattoni et al. (2016) [17]	Italy	Clinical Study	28 patients; 9 male, 19 female; aged 19–53	CAD/CAM porcelain laminate veneers	3D-Digital Smile System for virtual design and mock-up fabrication	None (pre-post evaluation)	Patient satisfaction with digital preview and mockup (VAS scale)	64.3% found digital preview very effective; 85.7% found mockup very effective; high patient satisfaction
Tallarico et al. (2025) [19]	Italy	Qualitative Study	5 patients; mean age 26.6; 4 female, 1 male	Feldspathic veneers after clear aligner therapy	exocad Smile Creator + TruSmile Video (AI-based 2D/4K simulation)	None (pre-post evaluation)	Smile aesthetics and function (0–10 scale), satisfaction with digital previews	Significant improvement in aesthetics (4.8→9.8) and function (6.6→9.4); AI preview rated higher than 2D; high overall satisfaction
Xuebo and Sutanto (2025) [18]	Indonesia	Mixed-Methods	Not specified (surveys: patients & dentists; interviews: 50 practitioners, 100 patients)	Not specified (aesthetic dental treatments utilizing DSD)	DSDApp, Smile Design Pro, CAD/CAM	Traditional smile design methods	Treatment precision, patient satisfaction (quantitative); communication, confidence, ease of use (qualitative)	DSD showed higher treatment precision (92% vs. 75%) and patient satisfaction (8.9/10 vs. 7.2/10); improved communication and confidence; challenges include cost and learning curve

The synthesized results from the included studies consistently demonstrate that DSD exerts a profoundly positive influence on patient-centered outcomes in esthetic restorative dentistry. This impact is primarily manifested through two interconnected themes: significantly enhanced patient satisfaction and empowerment through improved communication, and superior, predictable clinical and aesthetic results. Across diverse geographical contexts and utilizing various DSD methodologies, from basic 2D design to advanced 3D and AI-powered simulations, patients reported high levels of satisfaction.

The primary quantitative evidence comes from a randomized controlled trial by Luniyal et al., which provided robust, directly comparative data [7]. The DSD group demonstrated a statistically significant higher mean patient satisfaction score (87.2 ± 6.5) on a 0-100 VAS compared to the conventional methods group (81.5 ± 7.2). While the original paper did not report a precise p-value for this comparison, the reported means and standard deviations suggest a clinically meaningful difference. Furthermore, the DSD group had a significantly greater proportion of patients achieving "excellent" restoration outcomes (92% vs. 78%; outcomes encompassed fit, occlusion, and esthetics). Supporting these findings, observational studies reported high satisfaction levels using Likert scales. Sari et al. [12] reported mean satisfaction scores between 4.6 and 4.9 on a 5-point scale among 75 patients, with 92% of patients satisfied with their smile's appearance and 88% reporting improved self-confidence. The mixed-methods study by Xuebo and Sutanto [16] reported markedly higher scores for treatment precision (92% vs. 75%) and patient satisfaction (8.9/10 vs. 7.2/10) when DSD was employed. In a comparative analysis of different DSD conceptual approaches, Babaei et al. found high satisfaction with all three methods (Visagism, Proportional, and Stepwise Comprehensive) [16]. The Friedman test confirmed no statistically significant difference in overall satisfaction levels among the methods (p=0.094). Post-hoc analysis revealed no significant differences in satisfaction with tooth shape (p=0.859) or the general smile plan (p=0.309). A structured summary of the direction of effect for the primary outcome of patient satisfaction confirms the positive trend, as 6 out of 7 studies [7,12,16-19] reported clear positive findings for patient satisfaction and/or communication. One study was a supportive case report with high patient satisfaction at two-year follow-up [20]. This vote-counting approach reinforces the consistent, positive direction of the evidence, despite the heterogeneity.

A critical mechanism driving high satisfaction is DSD's ability to transform the patient-clinician relationship. Cattoni et al. found that 64.3% of patients considered the digital preview "very effective," and 85.7% found the physical mock-up "very effective" [17]. The technology mitigates anxiety by allowing patients to visually preview their proposed smile, fostering a sense of ownership and trust. Beyond subjective satisfaction, the DSD workflow contributes to superior objective clinical outcomes. Tallarico et al. noted that patients rated innovative AI-based previews even higher than traditional 2D simulations [19]. This process fosters a sense of ownership and trust. The meticulous digital planning facilitates more precise execution. Long-term follow-up data from Meereis et al. [20] and Sari et al. [12] affirm the durability of these results, showing excellent stability, marginal integrity, and color stability at two-year post-treatment, with only minor complications (e.g., veneer chipping in 5% of patients in the Sari et al. study [12]).

Discussion

This systematic review of seven studies found that the use of DSD in esthetic restorative dentistry is consistently associated with improved patient-centered outcomes - principally increased patient satisfaction, better esthetic perception, improved communication/decision-making, and frequently more predictable restorative results. The primary quantitative evidence comes from randomized and comparative studies showing higher satisfaction and superior clinical ratings for DSD workflows compared with conventional approaches. These findings align with broader patient-centered research, including the systematic review by Almasri et al., which outlines core constructs of orthodontic patient satisfaction and highlights the multidimensional nature of esthetic expectations and PROM variability [21]. This framework supports the interpretation that improvements observed with DSD relate not only to esthetics and function but also to patient perception, emotional response, and treatment involvement. Moreover, a randomized trial reported higher mean satisfaction scores and a larger proportion of “excellent” esthetic/functional outcomes in the DSD arm [7]. These trial results are in keeping with recent systematic and narrative reviews that conclude DSD improves communication, reduces errors and chair-time, and enhances patient acceptance and satisfaction [22]. Furthermore, evidence supporting the technical accuracy underpinning DSD has been strengthened by digital impression and model validation studies, including Jaber et al.’s intraoral scanning accuracy work [23], which confirms the reliability of digital datasets used for simulation, mock-up design, and CAD/CAM workflows. In addition to these clinical trial findings, foundational research on 3D imaging by Hajeer et al. [3] reinforces the methodological basis for DSD’s precision. The transition from 2D to fully integrated 3D and AI-driven simulations represents an important technological evolution, making patient-specific digital planning more predictable and better aligned with facially driven esthetic standards.

Two related mechanisms appear to drive the benefits. First, DSD provides a visual preview and concrete mock-up that aligns patient expectations with proposed treatment - reducing uncertainty and facilitating shared decisions. Multiple clinical reports and implementation studies describe how virtual mock-ups and physical mock-ups derived from DSD increase patient understanding and perceived predictability of results [24]. Second, the digital workflow (scanning, CAD, 3-D planning) enhances laboratory and clinical precision, which translates into improved fit, symmetry, and overall esthetic harmony in final restorations - objective improvements that reinforce patient satisfaction [17]. The positive effects of DSD were reported across a range of software platforms and complexity levels (from 2D photo planning to 3D CAD/CAM workflows and emerging artificial intelligence (AI)-augmented previews). Recent studies using AI-enhanced simulations reported especially high patient ratings for previews and significant gains in efficiency and aesthetic ratings, suggesting that advances in software may further amplify DSD’s benefits [19].

Despite generally favorable results, several limitations reduce the strength of inference. Included studies frequently used heterogeneous outcome measures (different satisfaction scales, timing of assessment, and varied psychosocial instruments), which complicates direct comparisons and precludes meta-analysis in this review. Many studies were observational, had small samples, or had short follow-up periods; although case reports and small cohorts report good one to two-year stability, robust long-term randomized data remain limited. A key methodological limitation is the absence of PROSPERO registration, which may reduce protocol transparency; however, all steps, including eligibility criteria, search strategy, and predefined outcomes, were fully documented to maintain reproducibility. Additionally, the review excluded non-English publications, which may have led to the omission of relevant evidence and introduced a potential language bias. Finally, implementation factors, including clinician experience, software choice, laboratory workflows, and patient selection, vary considerably and likely influence outcomes; these contextual differences were not always fully reported. Thus, current evidence indicates that DSD improves patient-centered outcomes in esthetic restorative dentistry, mainly by enhancing communication and treatment predictability. Yet higher-quality, standardized, and longer-term studies are needed to quantify these benefits across diverse clinical settings and to establish cost-effectiveness.

## Conclusions

This systematic review demonstrates that DSD significantly enhances patient-centered outcomes in esthetic restorative dentistry. Across diverse study designs and populations, DSD was consistently associated with improved patient satisfaction, esthetic perception, quality of life, and psychosocial well-being. Its ability to facilitate visual communication, personalize treatment planning, and provide accurate previews of expected results fosters trust, confidence, and greater involvement of patients in their care. Moreover, DSD contributes to predictable and durable clinical outcomes by improving treatment precision and efficiency compared to conventional methods. Despite these promising findings, the evidence base remains limited by heterogeneity in study designs, small sample sizes in several reports, and the relative scarcity of high-quality randomized controlled trials. Future research should prioritize well-designed longitudinal studies with standardized outcome measures to establish stronger evidence for the long-term effectiveness and cost-efficiency of DSD.

## Appendices

**Table 5:** Search strategies for included studies

Database/Register	Search String	Records
PubMed/MEDLINE	(“digital smile design”[All Fields] OR “DSD”[All Fields] OR “digital esthetic planning”[All Fields] OR “digital smile planning”[All Fields] OR (“esthetic dentistry”[MeSH Terms] AND “digital technology”[All Fields]) OR (“smile design”[All Fields] AND (“computer-aided design”[MeSH Terms] OR “CAD/CAM”[All Fields] OR “intraoral scanning”[All Fields])) AND (“patient satisfaction”[MeSH Terms] OR “patient-centered outcomes”[All Fields] OR “esthetic perception”[All Fields] OR “quality of life”[MeSH Terms] OR “OHRQoL”[All Fields] OR “psychosocial impact”[All Fields])	115
ScienceDirect	TITLE-ABSTR-KEY(“digital smile design” OR “DSD” OR “digital esthetic planning” OR “virtual smile design”) AND TITLE-ABSTR-KEY(“patient satisfaction” OR “esthetic outcome” OR “psychosocial” OR “quality of life”)	29
Cochrane Library	(“digital smile design” OR “digital smile planning” OR “DSD” OR “computer-aided esthetic planning”) AND (“satisfaction” OR “quality of life” OR “esthetic perception” OR “patient-centered”)	33
Google Scholar	“Digital Smile Design” AND (“esthetic dentistry” OR “restorative dentistry”) AND (“satisfaction” OR “quality of life” OR “patient-centered outcomes”)	168
